# The Management of the Cotyledonoid Leiomyoma of the Uterus: A Narrative Review of the Literature

**DOI:** 10.3390/ijerph18168521

**Published:** 2021-08-12

**Authors:** Francesca Buonomo, Sofia Bussolaro, Clarice de Almeida Fiorillo, Giorgio Giorda, Federico Romano, Stefania Biffi, Giuseppe Ricci

**Affiliations:** 1 Institute for Maternal and Child Health, I.R.C.C.S. “Burlo Garofolo”, 34137 Trieste, Italy; sofia.bussolaro@burlo.trieste.it (S.B.); clarice.dealmeidafiorillo@burlo.trieste.it (C.d.A.F.); federico.romano@burlo.trieste.it (F.R.); stefania.biffi@burlo.trieste.it (S.B.); giuseppe.ricci@burlo.trieste.it (G.R.); 2 Department of Medical, Surgical and Health Science, University of Trieste, 34127 Trieste, Italy; 3 Centro di Riferimento Oncologico di Aviano (C.R.O.), I.R.C.C.S., 33081 Aviano, Italy; ggiorda@cro.it

**Keywords:** cotyledonoid, leiomyoma, US, MRI

## Abstract

Cotyledonoid leiomyoma is an unusual uterine myoma due to some ultrasound features that mimic a malignant lesion facilitating the choice of radical surgery. This study aims to summarize the ultrasound and the magnetic resonance imaging aspects of this atypical lesion, and also discuss surgical treatment and pathological exam. It included all English case reports or case series until August 2021 found through PubMed, Google Scholar, and Scopus. A total of 94 cotyledonoid leiomyomas were reported, with a median tumor size of 12 cm. The typical ultrasound image is characterized by a large solid heterogeneous mass, with high vascularity, no shadowing, and indistinct margins within the myometrium. Magnetic resonance imaging shows the presence of merging isointense nodules to the myometrium in T1-weighted images, hyperintense in T2-weighted images, and contrast agent enhancement. Surgical treatment consists of hysterectomy (75 cases, 80%) or myomectomy (19 cases, 20%), without evidence of recurrence if complete. The placenta-like appearance observed during surgery supports this rare fibroid hypothesis. The intraoperative frozen section can be considered. Microscopically, no atypical cells, signs of mitotic activity or cell necrosis are found. To conclude, some preoperative and intraoperative aspects of this lesion are distinctive and may lead surgeons to opt for conservative surgery.

## 1. Introduction

Cotyledonoid leiomyoma is a rare variant of uterine myoma that macroscopically resembles placental cotyledons [1]. Its large reddish and spongiform nodulations, often protruding into the pelvic cavity, are typical of this form of myoma. The tongues of its tissue can also invade the myometrium by dividing it, giving this type of myoma the term “dissecting” (cotyledonoid dissecting leiomyoma) [2], raising the suspicion of a possible infiltrative pattern, despite its total benignity. Although the diagnosis may be entirely accidental because occasionally it can be mistaken for a typical form of fibroma, in some cases, it takes on extremely atypical features that may lead to the suspicion of malignancy [3]. In particular, specific clinical experiences which showed a large size of the lesion, its intense vascularization and heterogeneity, and its tendency to invade adjacent structures such as the broad ligament, have raised the suspicion of malignancy and have led surgeons to opt for radical surgery [3,4].

Histology is the most accurate method to reach a definitive diagnosis for cotyledonoid leiomyoma. So far, no example of malignant behavior or recurrence have been reported in the typical form of cotyledonoid leiomyoma. However, histological variants will have to be placed in the proper context for these patients’ optimal management. For instance, identifying variants with a more aggressive biological behavior and a potential vascular invasion tendency would be significant to guide the follow-up. Awareness of cotyledonoid leiomyoma variants is necessary for an accurate diagnosis and facilitates appropriate management decisions at the time of surgery. 

Interestingly, increasing evidence suggests that ultrasound and magnetic resonance imaging features, alongside the bulky mass placenta-like appearance during surgery, can substantially improve diagnosis quality. In particular, imaging evaluation can be an asset in managing patients who are appropriate candidates for fertility-sparing surgery and desire this approach. Indeed, imaging analysis would help evaluate the intrauterine tumor resection by myomectomy and the extrauterine tumor by excision. 

Based on a recently published case report series [3], whilst performing a more extensive study of the literature, this narrative review aims to briefly describe the distinctive aspects of this particular form of fibroid, considering not only imaging studies, but also pathological examination and hormonal and surgical treatments.

## 2. Material and Methods

An English literature search was conducted from inception until August 2021 in the following databases: PubMed (Medline), Google Scholar, and Scopus. “Cotyledonoid leiomyoma”, “cotyledonoid dissecting leiomyoma”, “grape-like myoma”, and “Sternberg tumor” were the search terms, and full-text articles were obtained to identify potentially eligible studies. Case studies and case series were included, while literature reviews and cases reported several times by the same authors in subsequent publications were excluded to avoid the same case being considered twice. Clinical and laboratory data were then studied, and the most significant imaging aspects were revised. Pelvic ultrasound and magnetic resonance imaging, surgical treatment, and pathological diagnosis were briefly discussed, based on all the available data. Limited data were achieved in most case reports’ ultrasound and resonance features; therefore, we reported all the cumulative characteristics described. 

## 3. Results and Discussion

A total of 94 cases with a histologically proven diagnosis of cotyledonoid leiomyoma (70 cases of cotyledonoid dissecting leiomyoma, 74.5%, and 24 of cotyledonoid leiomyoma, 25.5%) were collected, including our 13 cases recorded at the IRCCS Burlo Garofolo of Trieste and the Aviano Regional Cancer Centre [3].

**Figure 1:**
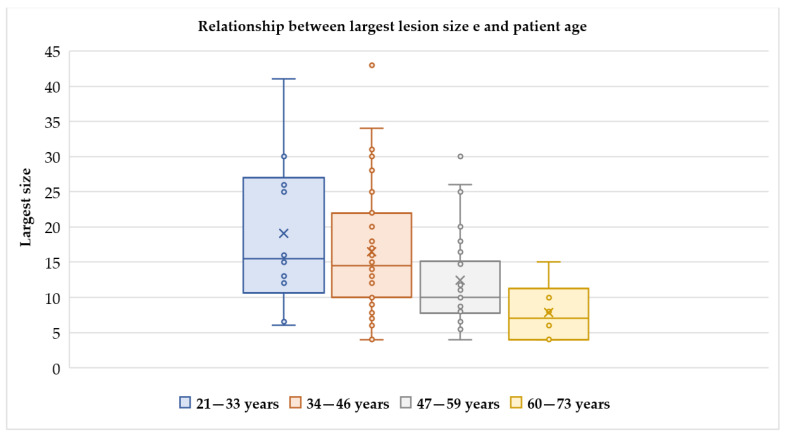
Relationship between the size of the lesion and patient age. It should be noted that the largest number of cases is in the 34–46 age group, when conservative surgery should be proposed due to the possible desire for pregnancy. With advancing age, the size of the lesion decreases, probably due to the effect of hormone depletion typical of the menopause.

The median age was 44 (range 21–73 years), covering the different ages of the women. Presenting symptoms were the same as for typical fibroids, such as the presence of abdominopelvic masses (38 cases, 40.4%), abnormal uterine bleeding (37 cases, 39.4%), abdominopelvic pain (12 cases, 12.8%), or abdominal swelling (3 cases, 3%), but cotyledonoid leiomyoma were also found incidentally (5 cases, 5.3%). Serum CA-125 was reported within normal limits or slightly increased [3]. Tumor size varied widely from 4 to 43 cm, with a median of 12 cm. The relationship between patient age and lesion size is described in Figure 1.

Many cases described in the literature report lesions located on the lateral wall of the uterus, although they can develop as a myoma in all uterine parts, even in the cervix and in the ovary. Because they often extend into the broad ligament and thus into the pelvic cavity, the suspicion of malignancy is even greater, especially in cases where the leiomyoma has a dissecting character. Table 1 summarizes all the cases described in this review with clinical symptoms, tumor size, pathological type, and therapy. 

### 3.1. Preoperative Assessment

Although considered benign tumors, the macroscopic appearance of these lesions associated with characteristic ultrasound features may induce malignancy suspicion.

**Figure 2:**
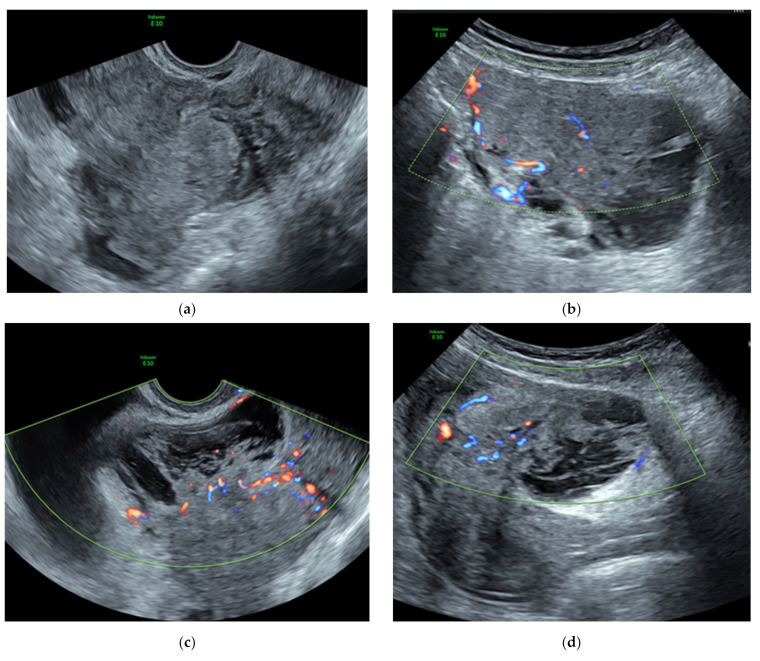

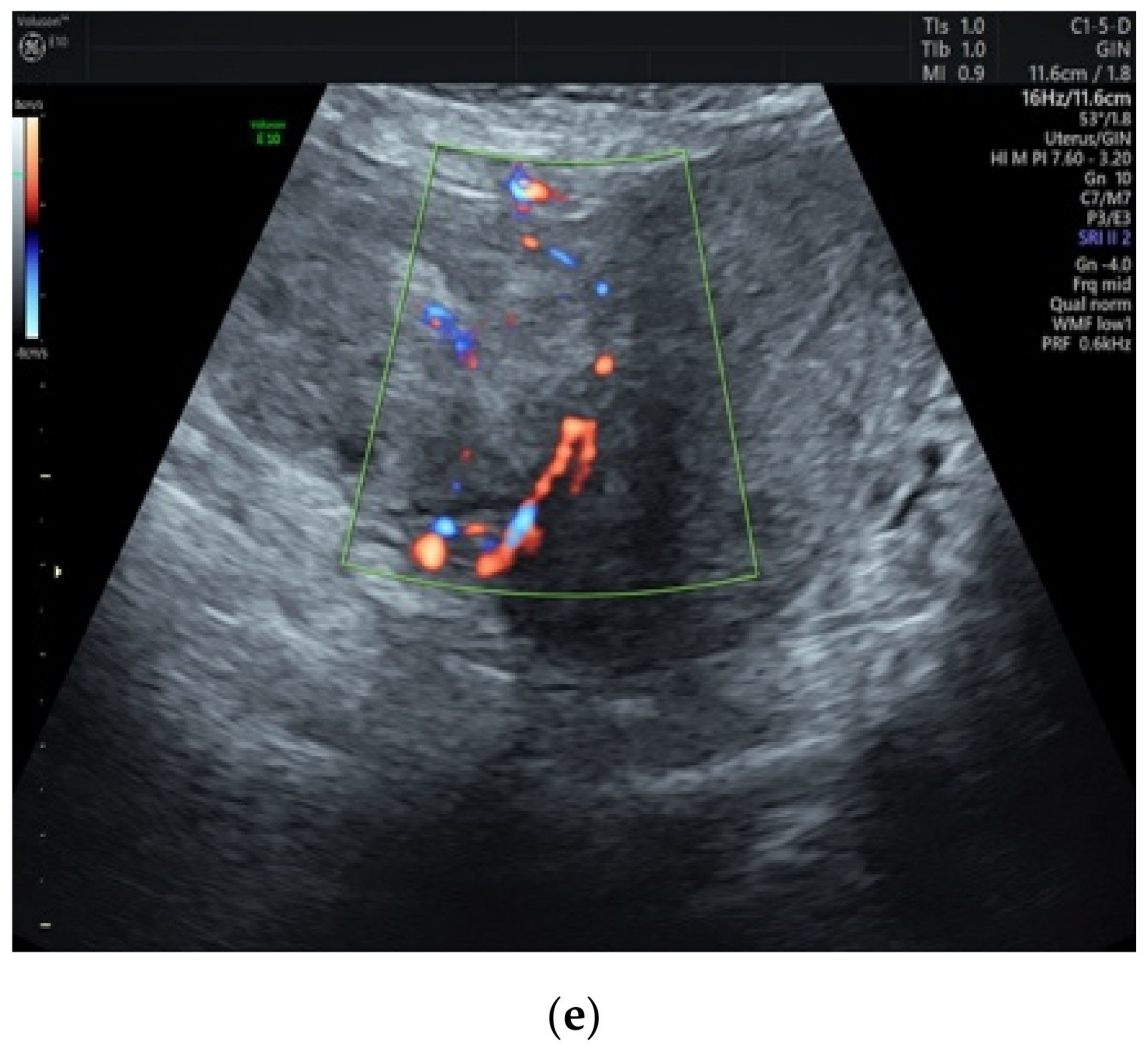
Uterine cotyledonoid dissecting leiomyoma and ultrasound features. Different US features of uterine cotyledonoid dissecting leiomyoma: note in (**a**) irregular margins and heterogeneous aspect of the mass; no evidence of capsule, no shadowing; (**b**) hypervascularization compared with the vascularity of the myometrium, color score 3; (**c**,**d**) the presence of colliquated areas; (**e**) note the uterus on the right side and the lesion on the left side emerging from the lateral wall of the uterine corpus. With the application of color doppler, the vascularization is visualized with color red and blue, in images (**b**–**e**).

The gynecologist can come across a heterogeneous irregular large mass with a high vascularization [4,42], no shadowing, and possible indistinct margins within the myometrium [3], described according to MUSA (the Morphological Uterus Sonographic Assessment) terms and definitions [60]. Most of these lesions are isoechoic compared to the myometrium and are surrounded by a capsule, which can be interrupted in the dissecting form. No sign of infiltration of pelvic organs, nor the parametrial involvement or the presence of ascites has been described in the typical form so far. Figure 2 shows some different features of ultrasound images of uterine cotyledonoid leiomyoma.

If required in the suspicion of infiltrative disease, pelvic magnetic resonance imaging can reveal a lobulated mass that shows isointensity to the myometrium in T1-weighted images, mostly hyperintensity in T2-weighted images, and contrast agent enhancement [3,4,26,34,41]. According to some authors [58], the appearance of the lesions in T2-weighted images can also be variable and more heterogeneous. On diffusion-weighted imaging, the lesion shows iso-signal intensity compared to the outer myometrium and the apparent diffusion coefficient map does not show restricted diffusion in the lesion [41].

### 3.2. Surgical and Hormone Therapies

**Figure 3:**
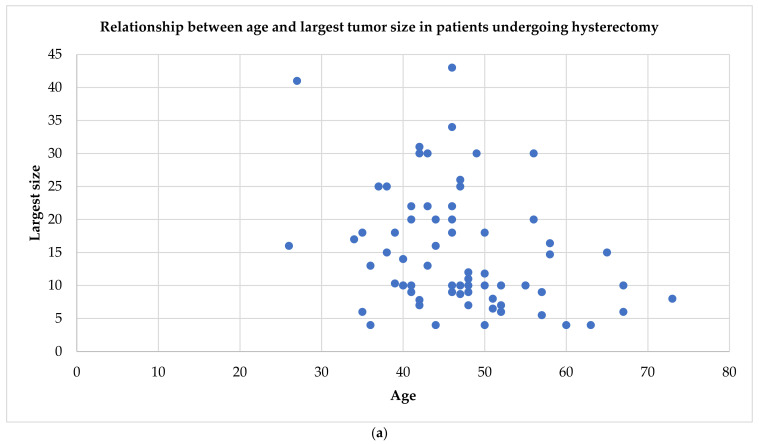

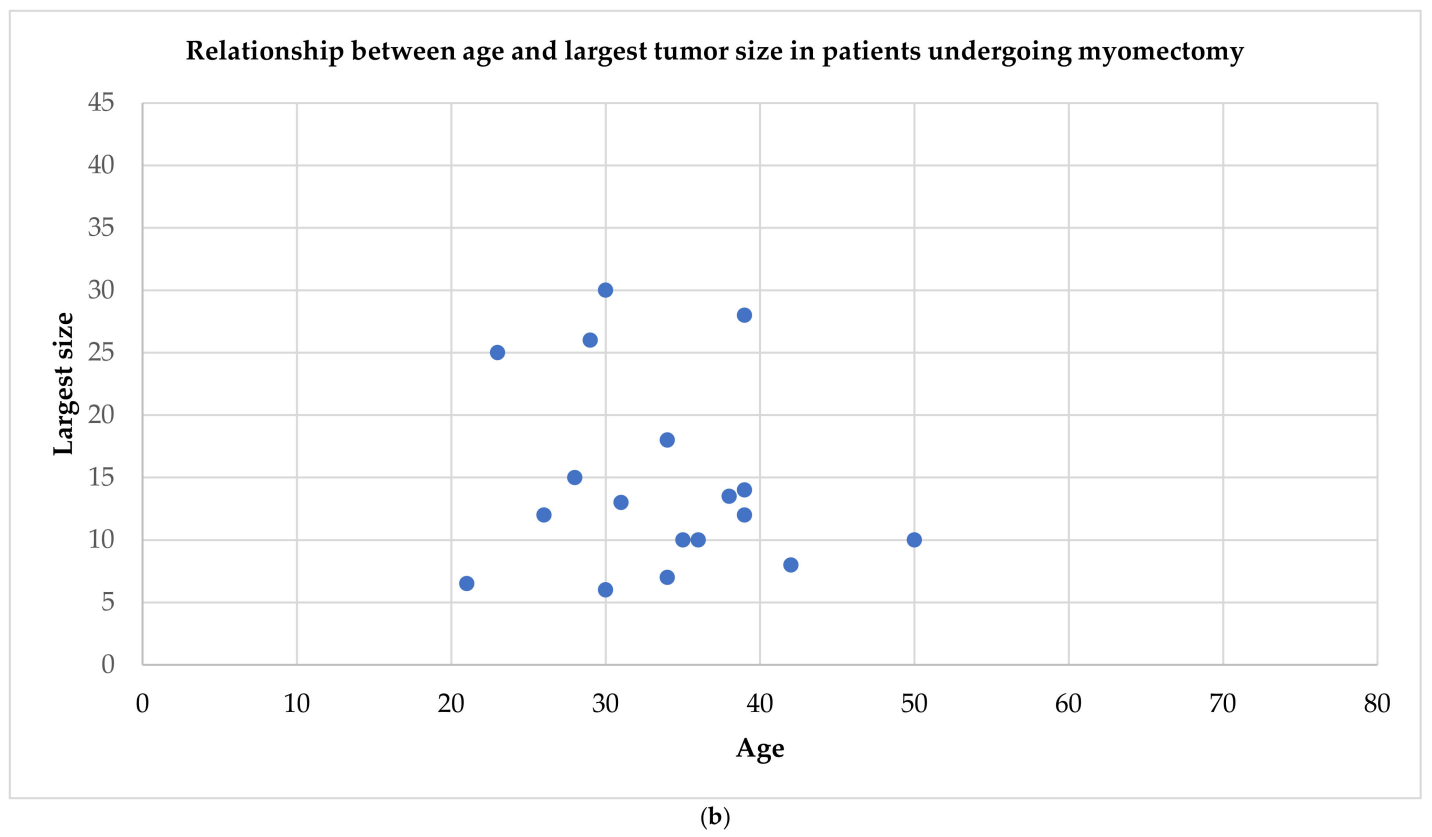
Relationship between age and tumor size in patients undergoing different surgical strategies. Almost all women between 40 and 50 years who have a cotyledonoid leiomyoma of 10–30 cm undergo hysterectomy (**a**). Conversely, for women of childbearing age between 20 and 40 years, myomectomy is attempted to save the uterus, regardless of the size of the tumor (even for sizes between 25 and 30 cm) (**b**).

Our data show that the majority of patients underwent a non-conservative treatment with a hysterectomy (80%), while simple tumor resection or myomectomy was carried out in only 19 patients (20%). The pre-surgical identification of a cleavage plan can guide the choice of the treatment. The cotyledonoid leiomyoma appears to have a capsule that creates the cleavage plane with the myometrium and thus effectively makes myomectomy possible. On the other hand, the capsule is present by ultrasound also in the cotyledonoid dissecting leiomyoma, but it is interrupted where it dissects the myometrium. Although myomectomy is easier in non-dissecting forms, even in the dissecting types it is feasible, regardless of the large size of the neoformation [1,10,45], as seen in Figure 3.

Hormone therapy with UPA (ulipristal acetate) or GnRH analogues has been described before myomectomy, resulting in improved symptoms and in reduction in mass volume [40,42], but available data are scarce and not sufficient to justify its use. Furthermore, as with typical fibroids, hormone therapy may change the consistency of the fibroid, making it less manageable and more prone to bleeding. Moreover, the cleavage plane would be difficult to identify during surgery. Not only is hormone therapy therefore not very popular with surgeons, but it is also limited by the possible side effects of the drug. Ulipristal acetate, in particular, has a limited use because it can cause acute liver failure. Furthermore, the use of hormone therapy in doubtful cases is not recommended. In these cases, in the face of suspicious preoperative aspects, we, therefore, reserve the right to complete the diagnostic course during surgery with direct visualization of the mass and possible frozen section.

Given the good outcome reported in cases treated with myomectomy, treatment options should be oriented to the simple resection of the tumor, especially in patients who are candidates for fertility-sparing surgery, while hysterectomy should only be considered if needed. Sometimes choosing minimally invasive surgery can be extremely difficult because the intraoperative macroscopic aspect and the preoperative ultrasound features may raise the suspicion of malignancy, inducing the surgeon to perform radical surgery.

If recognized, some preoperative and intraoperative elements may help to correctly identify the type of the lesion (Table 2). In situations of doubt, we recommend performing the extemporaneous intraoperative exam because if the diagnosis is confirmed, complete myomectomy alone is the treatment of choice [16,31,40,42]. In cases of incomplete excision, regrowth has also been described [2,31,50], motivating the need for a complete myomectomy even in the most conservative situations. If resection of the tumor is complete, follow-up at 2 years has been shown to be negative [3].

### 3.3. Differential Diagnosis

The differential diagnosis must consider benign pathologies (typical myoma, degenerated cystic myoma, cellular fibroid, and intravenous leiomyomatosis), and malignant ones. The comparison and differentiation of cotyledonoid leiomyoma from uterine sarcoma is the actual difficulty. Uterine sarcoma may have a rapid growth and ultrasound images can show an intense vascularization with irregular distribution of vessels and several anechoic areas due to cell necrosis [61]. The magnetic resonance imaging can detect irregular margins of the mass, presenting heterogeneous contrast enhancement areas with slightly high signal intensity on T1-weighted images in the site of cell necrosis [62]. According to some authors [63], the diffusion-weighted imaging and the apparent diffusion coefficient values measurement on magnetic resonance imaging could differentiate leiomyosarcomas from benign leiomyomas. High signal intensity on diffusion-weighted imaging and low apparent diffusion coefficient values seem to be typical of malignant tumors, due to the higher cell density. Nevertheless, although there are clinical signs and ultrasound and magnetic resonance features that tend to be primarily one or the other lesion, histology is the most accurate method to reach a definitive diagnosis.

### 3.4. Pathological Findings

The pathological study of the cotyledonoid leiomyoma reveals only benign aspects (Table 3). Despite the unusual macroscopic placenta-like appearance, the lesion is composed of benign uniform smooth muscle cells, rich in vessels. Macroscopically, the tumor consists of multiple congested reddish processes protruding on the uterine surface and sometimes with a dissecting character on the myometrium. No signs of malignancy appear under microscopy: no atypical cells, no signs of increased mitotic activity, no coagulating tumor necrosis. In only one case [47], symplastic features were detected, because of the presence of mononuclear and multinuclear atypical, bizarre cells. Despite these atypical microscopic features, no evidence of recurrence was observed after a 36 month follow-up.

Immunohistochemistry can confirm the benign leiomyomatosis nature of the lesion (Table 4). The tumor cells stain strongly positive with muscle-specific actin, desmin, vimentin, caldesmon, estrogen, and progesterone receptors, occasionally with CD10 (cluster of differentiation 10) [13,14,25,59]. An additional immunohistochemical marker used to differentiate between benign and malignant myometrial tumors is Bcl-2 (B-cell lymphoma 2), which prevents apoptotic cell death, promoting cellular replication. Normal myometrium shows negative to weak positive staining for Bcl-2. In leiomyoma, the Bcl-2 expression is more often and stronger than leiomyosarcoma and STUMP (smooth muscle tumors of uncertain malignant potential) [64]. According to some authors [65], the effect of this anti-apoptotic protein is enhanced by progesterone and leads to fibroid growth. In an immunohistochemical study by a Polish group [66] on four previously undescribed dissecting cotyledonoid leiomyomas, Bcl-2 was focally expressed in a single case and diffusely in the other three (a slight positivity was also reported in the manuscript of Shimizu et al. [43]). The whole immunohistochemical expression pattern (p16/Ki-67/bcl2-/WT-1/p53) was judged to be similar to that of usual type myomas and did not justify the macroscopic, placental-like appearance. Given the paucity of studies available on the cotyledonoid form, no major conclusions can be drawn. Additional new immunohistochemical markers, if identified, may help in doubtful cases.

### 3.5. Histological Variants

Cotyledonoid leiomyoma can have a variety of histological variants, the natures of which are not always predictable. In addition to the dissecting form, the following variants have been identified from the cases described so far: (1) 4 with intravenous leiomyomatosis [10], (2) 6 with intravascular growth [17,23,26,29,46,54], (3) 14 with hydropic degeneration [15,16,17,18,20,24,41,48,52,58], (4) 4 epithelioid variants [20,25,35], (5) 1 with adipocytic differentiation [38].

Among these forms, Niziurski et al. [58] described a case of cotyledonoid dissecting leiomyoma with prominent hydropic degeneration which seems to have developed such an aggressive character enough to infiltrate 7 cm into the walls of the small intestine. During surgery, the involved intestinal tract had a thickening of the serosa and was in adhesion with the grapelike lesion of cotyledonoid leiomyoma, so surgeons decided to remove it together with the lesion. Microscopic examination revealed only benign features, but a complete immunohistochemical stain was not performed. Again, no recurrences or metastasis were found. 

So far, only Kashima et al. in 2019 [54] have reported a case of cotyledonoid dissecting leiomyoma with intravascular growth (CD31 positive, which is not typical of cotyledonoid leiomyoma) and possible multiple lung metastases in a patient in whom there was evidence of round bilateral homogenous lung nodules by computed tomography imaging. The suspicion arose only from a cytological study of the smear specimen because the computed-tomography-guided needle biopsy of the lung lesion was not conclusive. Furthermore, after gynecological surgery alone, there was no subsequent dimensional increase in these lung lesions or other emerging lesions. For these reasons and the absence of follow-up at more than three months, it is not possible to make a conclusive diagnosis, but there seems to be some doubt that the intravascular component form may indeed be aggressive. However, in all other cases of cotyledonoid dissecting leiomyoma with intravascular growth, there was no evidence of metastases and the follow-up remained negative.

Non-neoplastic cystic lesions within the cotyledonoid dissecting leiomyoma were also reported (7 cases), including in cases associated with adenomyosis [43,46], endometriosis and endosalpingiosis [23,29], and adenoleiomyomatous components [10]. According to some authors [43], the dissecting nature of the cotyledonoid leiomyoma favors the incorporation into its wall of several parts of the myometrium, which can be already affected by adenomyosis or endosalgingiosis. However, it is not yet clear whether there is similar pathogenesis.

## 4. Conclusions

Knowledge of this unusual form of fibroid and its dissecting variant is crucial in managing the lesion, mainly as it avoids unnecessary extensive surgery. In our opinion, the most significant elements in the diagnosis of cotyledonoid leiomyoma are the typical ultrasound, magnetic resonance imaging features, and the placenta-like appearance of the bulky mass during surgery.

Further studies are needed to clearly define all variants of cotyledonoid leiomyoma, including their immunohistochemical expression. These insights will undoubtedly make it possible to comprehensively establish the possible vascular invasion tendency, allowing a more specific treatment for those variants.

## Figures and Tables

**Table 1:** Reported cases in the literature.

First Author	Year of Publication	Number of Cases Described	Age	Clinical Symptoms	Tumor Largest Size (cm) *	Tumor Volume (cm^3^)	Pathologic Type	Therapy
David [5]	1975	2	48; 65	AUB, P, uterine prolapse	12;15	NS	2 CDL	2 TH
Brand [6]	1995	1	24	Abdominal swelling	NS	NS	CDL	M
Roth [1]	1996	4	23–41	Pelvic mass, AUB	10–25 (25 cm)	NS	4 CDL	3 TH ± BSO, 1 M
Menolascino-Bratta [7]	1999	1	26	P	16	2240	CDL	TH + BSO + A
Roth [8]	2000	1	46	Menometrorrhagia	34	11560	CL	TH + BSO
Cheuk [9]	2002	1	55	Menorrhagia, uterine prolapse	10	360	CL	TH + BSO
Jordan [10]	2002	6	34–46	P, pelvic mass, menorrhagia	10–22 (18 cm)	NS	6 CDL (4 with intravenous leiomyomatosis)	1 M, 5 subtotal/TH ± BSO
Kim [11]	2002	1	26	AUB	12	NS	CDL	M
Stewart [12]	2003	1	58	Pelvic mass	16.4	1697.4	CDL	TH + BSO+O
Gurbuz [13]	2005	1	67	Abdominal mass	10	120	CL	TH + BSO
Saeed [14]	2006	1	27	P, constipation	41	13837.5	CDL	TH + BSO
Maimoon [15]	2006	1	40	Urinary retention	10	NS	CL with hydropic degeneration	TH + SO
Mathew [16]	2007	1	30	P in pregnancy	30	NS	CL with hydropic degeneration	M
Shelekhova [17]	2007	2	73;48	Pelvic mass, P	8;9	NS, 260	2 CDL (1 with intravascular growth, 1 with hydropic degeneration)	2 TH + BSO
Weissferdt [18]	2007	1	52	Menorrhagia, P	6	72	CDL with hydropic degeneration	TH + S
Özcimen [19]	2008	1	38	AUB, P	15	750	CDL	TH + BS
Misir [20]	2009	6	35–57	Pelvic mass, abdominal swelling, irregular menses, menorrhagia	5–30	NS	6 CDL (2 epithelioid, 5 with hydropic degeneration)	4 TH, 1 TH + BSO + O + A, 1 TH + SO
Preda [4]	2009	1	41	Incidental	9	NS	CDL	TH + ovariectomy
Adelipe [21]	2010	1	37	Menorrhagia	NS	NS	CDL	TH + O
Agarwal [22]	2010	1	52	Polymenorrhagia	10	NS	CDL	TH
Fukunaga [23]	2010	4a	35–56	Constipation, abdominal mass, hypermenorrhea, P	4–30	NS	4 CDLb	4 TH ± BSO
Aggarwal [24]	2011	1	52	Menorrhagia, uterine prolapse	10	NS	CL with hydropic degeneration	TH + SO
Soleymani [25]	2011	1	63	AUB, P	4	36	Epithelioid CDL	TH + BSO
Gezginig [26]	2011	1	57	P	9	162	CDL with intravascular growth	TH + BSO
Ersöz [27]	2011	1	51	Menorrhagia	8	NS	CL	TH + BSO
Kim [28]	2013	1	43	Pelvic mass and P	13	NS	CL	TH + BSO
Sellami [29]	2013	4c	47–52	Abdominal mass, hyper-menorrhea	7–30 (10 cm)	NS	4 CDLd	1 M, 3 subtotal/TH ± BSO
Bothale [30]	2013	1	39	Pelvic mass	18	4320	CL	TH
Roth [31]	2013	1	21	Menorrhagia	6.5	143	CDL	M, then TH + BSO
Makharoblidze [32]	2013	1	42	Menorrhagia, polymenorrhea, P	31	27900	CDL	TH
Onu [33]	2013	1	50	Chronic lower back pain	4	30	CDL	RH + BSO + O + A
Tanaka [34]	2013	1	36	Incidental	10	NS	CDL	M
Chawla [35]	2014	1	42	Dysmenorrhea, P	7	245	Epithelioid CL	TH + BSO
Meena [36]	2014	1	40	P	14	NS	CDL	TH + BSO
Geynisman [37]	2014	1	50	P	18	1890	CDL	TH + BSO
Blake [38]	2014	1	56	AUB	20	NS	CDL with adipocytic differentia-tion	RH + BSO + O
Bas [39]	2015	1	46	P, menometrorrhagia	22	5280	CL	TH
Saeki [40]	2015	2	44; 31	Incidental, P	20;13	NS, M **: 497.3	2 CDL	1 TH + BSO, 1 GnRHanalogs + M
Motoshima [41]	2016	1	39	Abdominal mass	14	1176	CDL with hydropic degeneration	M
Raga [42]	2016	1	28	Menorrhagia, dysmenorrhea	15	1435.4	CDL	UPA + M
Shimizu [43]	2016	1	40	Menorrhagia	10	810	CDL	TH + BS
Xu [44]	2016	4	37–55	Pelvic mass	7–30	NS, 12000, 5250, NS	4 CDL	4 TH ± BSO
Buckshee [45]	2017	1	29	P	26	6760	CDL	M
Merchant [46]	2017	1	35	Dysmenorrhea and menorrhagia	10	210	CDL with intra-vascular growth	M
Sonmez [47]	2017	1	38	P	13.5	1275.8	CL with symplastic features	M
Rahman [48]	2018	1	46	P, abdominal swelling	43	31648	CDL with hydropic degeneration	TH + SO
Smith [49]	2018	1	42	Pelvic vaginal cuff mass	8	240	CDL	Resection
Khatun [50]	2018	1	48	P	10	320	CDL	TH + BSO
Rocha [51]	2018	1	38	Menorrhagia, P	25	13800	CDL of the uterus and the ovary	TH + BSO
Tuli [52]	2018	1	50	Menorrhagia	11.8	681.5	CDL with hydropic degeneration	TH + BSO
Jamal [53]	2019	1	60	P	4	NS	CDL	TH
Kashima [54]	2019	1	43	Menorrhagia	22	1694	CDL with intravascular growth	TH + BSO
Özdemir [55]	2019	1	34	Menorrhagia, P	17	NS	CDL	TH
Lenz [56]	2020	1	64	P	NS	NS	CDL	TH + R
Parker [57]	2020	1	39	Abdominal mass	28	NS	CL	M
Niziurski [58]	2020	1	41	AUB, pelvic mass	20	5100	CDL with hydropic degeneration	TH + BSO + intestine resection
Buonomo [3]	2020	13	30–67	Pelvic mass, P, incidental, metrorrhagia	4–14,7	489.6, 280, 132.8, 305, 770, 480, 210, 49, 165, 1411 M **: 157.5, 106.5, 1188	3 CDL, 10 CL	3 M, 4 TH + BSO, 2 TH + BS, 1 TH + S, 2 RH + BSO, 1 RH + S + O

Abbreviations: CDL—cotyledonoid dissecting leiomyoma; CL—cotyledonoid leiomyoma; AUB—abnormal uterine bleeding; P—abdominopelvic pain; NS—not specified; TH—total hysterectomy; M—myomectomy; BSO—bilateral salpingo-oophorectomy; SO—unilateral salpingo-oophorectomy; UPA—ulipristal acetate, BS—bilateral salpingectomy; A—appendicectomy; RH—radical hysterectomy; O—omentectomy; S—unilateral salpingectomy; R—unilateral parametrectomy, partial resection of the bladder wall and resection of the distal part of the ureter. * The maximum size of the lesion undergoing myomectomy is indicated in brackets if volume calculation is not available. ** Volumes of masses treated by myomectomy. a: In these four cases reported, a case already described in 1998 by the same author [23] is included, which has therefore been excluded from our review. b: 3 with perinodular hydropic change, 2 with intravascular growth, 1 with endometriosis, and 1 with endosalpingiosis; c: In these four cases reported, a case already described by Driss et al. in 2008 [59] is included, which has been therefore excluded from our review. d: 3 with perinodular hydropic change, 1 with intravascular growth, 1 with endometriosis, and 1 with endosalpingiosis.

**Table 2:** Key points when considering a cotyledonoid leiomyoma.

Pelvic Ultrasound	Magnetic Resonance Imaging	During Surgery
Bulky uterus due to a solid heterogeneous mass High vascularity (color score > 3) No shadowing Possible indistinct/irregular margins within the myometrium	Isointensity to the myometrium in T1-weighted images Hyperintensity in T2-weighted images Contrast agent enhancement Iso-signal intensity in diffusion-weighted imaging No restricted diffusion in apparent diffusion coefficient map	Placenta-like appearance Consider the extemporaneous intraoperative exam

**Table 3:** The pathological features of cotyledonoid leiomyoma.

Gross Pathology	Microscopic Elements
Placenta-like appearance Congested and reddish mass Multiple exophytic processes Possible dissection into the myometrium	Fascicles of smooth muscle cells Rich vascular component No atypical cells, no increased mitotic activity, no coagulating tumor necrosis

**Table 4:** The immunohistochemistry of cotyledonoid leiomyoma.

Positivity [9,20]	Negativity [25]
SMA Desmin Vimentin Caldesmon Estrogen receptors Progesterone receptors HHF-35 Occasionally CD10	CD117 S100 Melanocyte Specific Antigen Melan A CAM 5.2 MNF 116 EMA Renal cell carcinoma antigen Calretinin CK5/6 CD68 Inhibin

Abbreviations: SMA—smooth muscle actin; HHF—35-anti-muscle actin antibody; CD—cluster of differentiation; S—Schwann cell marker; CAM—cytokeratin antibody marker; MNF—anti-cytokeratin antibody; EMA—epithelial membrane antigen; CK—cytokeratin.

## Data Availability

The authors confirm that the data supporting the findings of this study are available within the article.

## References

[1] Roth L.M., Reed R.J., Sternberg W.H. (1996). Cotyledonoid Dissecting Leiomyoma of the Uterus. the Sternberg Tumor. Am. J. Surg. Pathol..

[2] Smith C.C., Gold M.A., Wile G., Fadare O. (2012). Cotyledonoid Dissecting Leiomyoma of the Uterus: A Review of Clinical, Pathological, and Radiological Features. Int. J. Surg. Pathol..

[3] Buonomo F., Bussolaro S., Giorda G., Romano F., Biffi S., Ricci G. (2020). Cotyledonoid Leiomyoma Clinical Characteristics, Imaging Features, and Review of the Literature. J. Ultrasound Med..

[4] Preda L., Rizzo S., Gorone M.S., Fasani R., Maggioni A., Bellomi M. (2009). MRI Features of Cotyledonoid Dissecting Leiomyoma of the Uterus. Tumori J..

[5] David M.P., Homonnai T.Z., Deligdish L., Loewenthal M. (1975). Grape-Like Leiomyomas of the Uterus. Int. Surg..

[6] Brand A.H., Scurry J.P., Planner R.S., Grant P.T. (1995). Grapelike Leiomyoma of the Uterus. Am. J. Obstet. Gynecol..

[7] Menolascino-Bratta F., Garcia de Barriola V., Naranjo de Gomez M., Garcia Tamayo J., Suarez J.A., Hernandez Chacon A.V. (1999). Cotyledonoid Dissecting Leiomyoma (Sternberg Tumor): An Unusual Form of Leiomyoma. Pathol. Res. Pract..

[8] Roth L.M., Reed R.J. (2000). Cotyledonoid Leiomyoma of the Uterus: Report of a Case. Int. J. Gynecol. Pathol..

[9] Cheuk W., Chan J.K., Liu J.Y. (2002). Cotyledonoid Leiomyoma: A Benign Uterine Tumor with Alarming Gross Appearance. Arch. Pathol. Lab. Med..

[10] Jordan L.B., Al-Nafussi A., Beattie G. (2002). Cotyledonoid Hydropic Intravenous Leiomyomatosis: A New Variant Leiomyoma. Histopathology.

[11] Kim M.J., Park Y.K., Cho J.H. (2002). Cotyledonoid Dissecting Leiomyoma of the Uterus: A Case Report and Review of the Literature. J. Korean Med. Sci..

[12] Stewart K.A., Ireland-Jenkin K., Quinn M., Armes J.E. (2003). Cotyledonoid Dissecting Leiomyoma. Pathology.

[13] Gurbuz A., Karateke A., Kabaca C., Arik H., Bilgic R. (2005). A Case of Cotyledonoid Leiomyoma and Review of the Literature. Int. J. Gynecol. Cancer.

[14] Saeed A.S., Hanaa B., Faisal A.S., Najla A.M. (2006). Cotyledonoid Dissecting Leiomyoma of the Uterus: A Case Report of a Benign Uterine Tumor with Sarcomalike Gross Appearance and Review of Literature. Int. J. Gynecol. Pathol..

[15] Maimoon S., Wilkinson A., Mahore S., Bothale K., Patrikar A. (2006). Cotyledonoid Leiomyoma of the Uterus. Indian J. Pathol. Microbiol..

[16] Mathew M., Gowri V., Al Hamdani A., Machado L., Rao K., Shabnam S. (2007). Cotyledonoid Leiomyoma in Pregnancy. Obstet. Gynecol..

[17] Shelekhova K.V., Kazakov D.V., Michal M. (2007). Cotyledonoid Dissecting Leiomyoma of the Uterus with Intravascular Growth: Report of Two Cases. Virchows Arch..

[18] Weissferdt A., Maheshwari M.B., Downey G.P., Rollason T.P., Ganesan R. (2007). Cotyledonoid Dissecting Leiomyoma of the Uterus: A Case Report. Diagn. Pathol..

[19] Özçimen E.E., Kıyıcı H., Uçkuyu A., Akyürek C. (2008). Cotyledonoid Dissecting Type Leiomyoma of the Uterus: A Case Report and Review of the Literature. Gynecol. Obstet. Reprod. Med..

[20] Misir A., Daya D., Sur M. (2009). Cotyledonoid Dissecting Leiomyoma of the Uterus (Sternberg Tumour): A Clinicopathological Study of Six Cases. Can. J. Pathol..

[21] Adedipe T.O., Vine S.J. (2010). Dissecting Cotyledonoid Leiomyoma: A Rare Cause of Chronic Intractable Menorrhagia (Not Amenable to Medical Treatment). Case Report. Eur. J. Gynaecol. Oncol..

[22] Agarwal R., Radhika A., Malik R., Radhakrishnan G. (2010). Cotyledonoid Leiomyoma and Non-Descent Vaginal Hysterectomy. Arch. Gynecol. Obstet..

[23] Fukunaga M., Suzuki K., Hiruta N. (2010). Cotyledonoid Dissecting Leiomyoma of the Uterus: A Report of Four Cases. Am. J. Surg. Pathol..

[24] Aggarwal S., Arora V.K. (2011). An Unusual Variant of Leiomyoma Masquerading Peroperatively as Sarcoma. Indian J. Cancer.

[25] Soleymani Majd H., Ismail L., Desai S.A., Reginald P.W. (2011). Epithelioid Cotyledonoid Dissecting Leiomyoma: A Case Report and Review of the Literature. Arch. Gynecol. Obstet..

[26] Gezginc K., Yazici F., Selimoglu R., Tavli L. (2011). Cotyledonoid Dissecting Leiomyoma of the Uterus with Intravascular Growth in Postmenopausal Woman: A Case Presentation. Int. J. Clin. Oncol..

[27] Ersoz S., Turgutalp H., Mungan S., Guvendi G., Guven S. (2011). Cotyledonoid Leiomyoma of Uterus: A Case Report. Turk. J. Pathol..

[28] Kim N.R., Park C.Y., Cho H.Y. (2013). Cotyledonoid Dissecting Leiomyoma of the Uterus with Intravascular Luminal Growth: A Case Study. Korean J. Pathol..

[29] Sellami R., Nasfi A., Doghri R., Nesrine M., Sassi S., Charfi L., Mrad K., Romdhane K.B. (2013). Cotyledonoid Dissecting Leiomyoma of the Uterus: A Report of Four Cases. J. Gynecol. Surg..

[30] Bothale A.A., Bothale K.A., Mahore S.D., Wilkinson A.R. (2013). Case Report: Cotyledonoid Leiomyoma. J. Basic Clin. Reprod. Sci..

[31] Roth L.M., Kirker J.A., Insull M., Whittaker J. (2013). Recurrent Cotyledonoid Dissecting Leiomyoma of the Uterus. Int. J. Gynecol. Pathol..

[32] Makharoblidze E., Goishvili N., Mchedlishvili M., Khakhutaishvili I., Jangavadze M. (2013). Unusual Types of Smooth Muscle Tumors of Uterine Corpus: Case Reports and Literature Review. Georgian Med. News.

[33] Onu D.O., Fiorentino L.M., Bunting M.W. (2013). Cotyledonoid Dissecting Leiomyoma as a Possible Cause of Chronic Lower Back Pain. BMJ Case Rep..

[34] Tanaka H., Toriyabe K., Senda T., Sakakura Y., Yoshida K., Asakura T., Taniguchi H., Nagao K. (2013). Cotyledonoid Dissecting Leiomyoma Treated by Laparoscopic Surgery: A Case Report. Asian J. Endosc. Surg..

[35] Chawla I., Bhardwaj M., Sareen N., Khattar N. (2014). Epithelioid Cotyledonoid Leiomyoma of Uterus. BMJ Case Rep..

[36] Meena L.N., Aggarwal A., Jain S. (2014). Cotyledonoid Leiomyoma of Uterus. J. Obstet. Gynaecol. India.

[37] Geynisman J., Pagan C., Pirog E., Holcomb K. (2014). Cotyledonoid Dissecting Leiomyoma. Int. J. Gynecol. Obstet..

[38] Blake E.A., Cheng G., Post M.D., Guntupalli S. (2014). Cotyledonoid Dissecting Leiomyoma with Adipocytic Differentiation: A Case Report. Gynecol. Oncol. Rep..

[39] Bas S., Selcuk I., Sirvan L., Yalcin H., Gungor T. (2015). Cotyledonoid Myoma: A Distinct Entity and a Diagnostic Dilemma in Gynecology. J. Cases Obstet. Gynecol..

[40] Saeki H., Suzuki C., Yamasaki S., Hashizume A., Izumi H., Suzuki F., Ishi K., Nojima M., Hino O. (2015). Cotyledonoid Dissecting Leiomyoma of the Uterus: Report of Two Cases. Arch. Gynecol. Obstet..

[41] Motoshima S., Irie H., Nakazono T., Yamasaki F., Nakao Y. (2016). Mri Findings of Cotyledonoid Dissecting Leiomyoma of the Uterus. Pak. J. Radiol..

[42] Raga F., Cholvi S., Pascual C., Boigues D., Sanchez C., Cano A. (2016). More to be Learned about Cotyledonoid Dissecting Leiomyoma. Ultrasound Int. Open.

[43] Shimizu A., Tanaka H., Iwasaki S., Wakui Y., Ikeda H., Suzuki A. (2016). An Unusual Case of Uterine Cotyledonoid Dissecting Leiomyoma with Adenomyosis. Diagn. Pathol..

[44] Xu T., Wu S., Yang R., Zhao L., Sui M., Cui M., Chang W. (2016). Cotyledonoid Dissecting Leiomyoma of the Uterus: A Report of Four Cases and a Review of the Literature. Oncol. Lett..

[45] Buckshee K., Harsh R., Deepshikha A., Tanya R.B. (2017). A Bizarre Highly Vascular Tumor with Alarming Presentation: A Diagnostic Dilemma. Int. J. Reprod. Contracept. Obstet. Gynecol..

[46] Merchant K., Chern B., Chew S.H. (2017). Cotyledonoid Dissecting Leiomyoma with Intravascular Growth Pattern and Intra-Tumoural Endometrial Glands and Stroma: A Case Report. Case Report. Clin. Pathol..

[47] Sonmez F.C., Tosuner Z., Karasu A.F.G., Arici D.S., Dansuk R. (2017). Cotyledonoid Dissecting Leiomyoma with Symplastic Features: Case Report. Rev. Bras. Ginecol. Obstet..

[48] Rahman H., Dubey S., Chavan P., Sherpa M.L., Sharma B.K., Khalda E. (2018). Perinodular Hydropic Degeneration in a Case of Leiomyoma Uterus: A Rare Case Report and Review of Literature. Indian Obstet. Gynaecol..

[49] Smith H., Jung N., Carter A., Watson M., Singh A. (2018). Post-Hysterectomy Extrauterine Cotyledonoid Leiomyoma in a 42-Year-Old Female. Urol. Case Rep..

[50] Khatun S.F., Nazneen T., Khatun S. (2018). A 48 Year Old Postmenopausal Woman with Dull Aching Lower Abdominal Pain and Heaviness in the Abdomen. Bangabandhu Sheikh Mujib Med. Univ. J..

[51] Rocha A.C., Oliveira M., Luis P., Nogueira M. (2018). Cotyledonoid Dissecting Leiomyoma of the Uterus: An Unexpected Diagnosis after Delivery. Acta Med. Port..

[52] Tuli A.G., Goyal S. (2018). Cotyledonoid Dissecting Leiomyoma of the Uterus. J. Datta Meghe Inst. Med. Sci. Univ..

[53] Jamal I., Gupta R.K., Sinha R.K., Bhadani P.P. (2019). Cotyledonoid Dissecting Leiomyoma: An Uncommon Form of a Common Disease. Obstet. Gynecol. Sci..

[54] Kashima J., Tonooka A., Taguchi A., Funata N., Yasugi T., Hishima T. (2019). A Cotyledonoid Dissecting Leiomyoma with an Intravascular Component and Adenomyosis Accompanied with Possible Multiple Lung Metastases: A Case Report. Hum. Pathol. Case Rep..

[55] Ozdemir O., Sagır G., Akbas B., Guven S., Reis A. (2019). A Case Report on Recurrent Cotyledonoid Dissecting Leiomyoma. J. Clin. Obstet. Gynecol..

[56] Lenz J., Chvátal R., Konečná P. (2020). Dissecting Leiomyoma of the Uterus with Unusual Clinical and Pathological Features. Ceska Gynekol..

[57] Parker W.H., Turner R., Schwimer S., Foshag L. (2020). Massive Cotyledenoid Leiomyoma Treated with Uterine-Conserving Surgery. F&S Rep..

[58] Niziurski P., Roszkowska-Purska K., Młodawski J., Młodawska M., Malmur M. (2020). Cotyledonoid Dissecting Leiomyoma of the Uterus with Infiltration of Tumor Cells within Intestinal Wall: The First Polish Case Report of a Rare Uterine Tumor and Review of the Literature. Med. Stud. Stud. Med..

[59] Driss M., Zhioua F., Doghri R., Mrad K., Dhouib R., Romdhane K.B. (2009). Cotyledonoid Dissecting Leiomyoma of the Uterus Associated with Endosalpingiosis. Arch. Gynecol. Obstet..

[60] Van den Bosch T., Dueholm M., Leone F.P., Valentin L., Rasmussen C.K., Votino A., Van Schoubroeck D., Landolfo C., Installe A.J., Guerriero S. (2015). Terms, Definitions and Measurements to Describe Sonographic Features of Myometrium and Uterine Masses: A Consensus Opinion from the Morphological Uterus Sonographic Assessment (MUSA) Group. Ultrasound Obstet. Gynecol..

[61] Ludovisi M., Moro F., Pasciuto T., Di Noi S., Giunchi S., Savelli L., Pascual M.A., Sladkevicius P., Alcazar J.L., Franchi D. (2019). Imaging in Gynecological Disease (15): Clinical and Ultrasound Characteristics of Uterine Sarcoma. Ultrasound Obstet. Gynecol..

[62] Kido A., Togashi K., Koyama T., Yamaoka T., Fujiwara T., Fujii S. (2003). Diffusely Enlarged Uterus: Evaluation with MR Imaging. Radiographics.

[63] Tamai K., Koyama T., Saga T., Morisawa N., Fujimoto K., Mikami Y., Togashi K. (2008). The Utility of Diffusion-Weighted MR Imaging for Differentiating Uterine Sarcomas from Benign Leiomyomas. Eur. Radiol..

[64] Rubisz P., Ciebiera M., Hirnle L., Zgliczyńska M., Łoziński T., Dzięgiel P., Kobierzycki C. (2019). The Usefulness of Immunohistochemistry in the Differential Diagnosis of Lesions Originating from the Myometrium. Int. J. Mol. Sci..

[65] Matsuo H., Maruo T., Samoto T. (1997). Increased Expression of Bcl-2 Protein in Human Uterine Leiomyoma and its Up-Regulation by Progesterone. J. Clin. Endocrinol. Metab..

[66] Zielinska-Pajak E., Liszka L., Pajak J., Golka D. (2010). Immunohistochemical Profile of Cotyledonoid Dissecting Leiomyoma of the Uterus. Virchows Arch..

